# B-1 Cell Heterogeneity and the Regulation of Natural and Antigen-Induced IgM Production

**DOI:** 10.3389/fimmu.2016.00324

**Published:** 2016-09-09

**Authors:** Nicole Baumgarth

**Affiliations:** ^1^Department of Pathology, Microbiology and Immunology, Center for Comparative Medicine, University of California Davis, Davis, CA, USA

**Keywords:** natural IgM, B-1 cells, B cell development, immune regulation, innate-like lymphocytes

## Abstract

A small subset of B cells, termed B-1 cells, with developmental origins, phenotypes, and functions that are distinct from those of conventional B cells exist in mice. It contributes the vast majority of spontaneously produced “natural” IgM. Natural IgM is constitutively produced, even in the absence of microbiota, and fulfills many distinct functions in tissue homeostasis and host defense. B-1 cells also respond with IgM production to innate signals and pathogen exposure, while maintaining steady-state levels natural IgM. Thus, within the B-1 cell pool, cells of distinct and heterogeneous functionality must exist to facilitate these different functions. This review considers three factors that may contribute to this heterogeneity: first, developmental differences regarding the origins of the precursors, second, tissue-specific signals that may differentially affect B-1 cells in the tissue compartments, and finally responsiveness to self-antigens as well as innate and antigen-specific signals. All three are likely to shape the repertoire and responsiveness of B-1 cells to homeostatic- and antigen-induced signals and thus contribute to the functional heterogeneity among these innate-like B cells.

## Introduction

The B cell compartment of all jawed vertebrates contains populations of spontaneous “natural” Ig-secreting cells (1). These antibodies are broadly self-reactive and most are of the IgM isotype. The best-studied natural antibody-producing cell population is that of mice, in which a subset of CD5-expressing B cells, CD5 was thought of until then as a T cell-restricted surface receptor, was linked to natural IgM production (2). Further studies showed differences in B-1 cell development origins, tissue distribution, and responsiveness to antigens and mitogens compared with classical follicular B cells and thus identified them as distinct from the majority “conventional” B cell population [reviewed in Ref. (3, 4)]. Because these cells appear earlier in ontogeny than conventional B cells, they were termed “B-1 cells” and conventional B cells “B-2 cells”; CD5 expression is used to differentiate B-1 cells further into B-1a (CD5^+^) and B-1b (CD5^−^).

Numerous groups demonstrated that in addition to generating natural antibodies, B-1 cells also actively contribute to pathogen-induced immune responses (2). Explored have been responses to *Francisella* spp (5, 6), *Borrelia hermsii* (7–9), *Salmonella typhi* (10, 11), *Streptococcus pneumoniae* (12–14), and influenza virus (15–17). In each case, the response consisted of increased B-1 cell-derived IgM production, measured in regional lymph nodes, the spleen, and/or in serum. This raises important questions about the regulation of natural versus antigen-induced antibody production by B-1 cells. Studies on influenza virus infection showed that despite an increased local production of B-1 cell-derived IgM, natural serum IgM levels remained unaffected (15), suggesting the presence of distinct subsets of B-1 cells that contribute systemic natural and enhanced infection-induced local IgM production, respectively.

At least two non-mutually exclusive models may explain these observations: a “division of labor” model, as proposed (14), in which distinct B-1 cell subsets exist, some responsible for natural antibody production. In the studies by Haas et al., B-1b cells responded to *S. pneumoniae* antigens by making antibodies, whereas B-1a cells constitutively produced natural IgM antibodies against other components of *S. pneumoniae*. However, given that in other infections (see below), B-1a cells were also shown to respond with increased antibody production, a simple division of labor between B-1a and B-1b seems unlikely. Another model is that the degree of self-antigen-mediated stimulation of the BCR and/or additional costimulatory signals may support a certain number of B-1 cells to differentiate to natural antibody-producing cells, while others remain quiescent, until stimulation by innate signals and/or antigen exposure activates these cells above a certain threshold required for their differentiation. Common to both models is the idea that the B-1 cell pool contains B-1 cells of different functional properties.

The literature supports the notion of a heterogeneous B-1 cell pool, but the causes for this heterogeneity are largely unknown and little explored. Below, we consider three factors that may modulate B-1 cell functions: (1) the multiple developmental origins of B-1 cells, (2) tissue-specific signals, and (3) differences in exposure to and responsiveness of B-1 cells to self- and foreign antigens. Determining the relative impact of these signals on the functionality of B-1 cells could clarify much of the biology of this cell population and of one of its important products: natural IgM.

## Multiple Developmental Origins Contribute to the B-1 Cell Pool

Early studies demonstrated that B cell precursors exist in the splanchnopleura of the developing mouse embryo, which give rise only to B-1 but not B-2 cells, but the precursors were not identified (18). More recent reports supported these findings by showing that the earliest B-1 cells arise from extra-hematopoietic sources, the blood island cells of the yolk sac (19, 20), similar to the development of some macrophage populations (21). In 2006, Montecino-Rodriguez et al. identified B cell precursors in fetal liver and in small numbers also in the bone marrow of adult mice that give rise only to B-1 cells (22). These B-1 cell-restricted precursors (pre–pro-B) among otherwise “lineage negative cells” were differentiated from early conventional (B-2) cell precursors by their lack of CD45R (B220) and strong expression of CD19. While present also in the adult bone marrow, it was further reported that by 3–6 weeks after birth, B-1 cell precursors are no longer contributing significantly to the adult B-1 cell pool (23), consistent with a previous body of literature that showed B-1 cells to be a mainly fetal- and neonatal-developed population that is maintained by self-renewal rather than *de novo* development (24, 25).

It appears that the bone marrow precursors can be activated in situations of severe lymphopenia, however, as occurs following adoptive cell transfer of bone marrow into lethally irradiated recipients (26, 27). In that situation, the emerging B-1 cell populations are much more heavily skewed toward CD5^−^ B-1b cell development. The reasons for this remain to be explored. Thus, existing data support the concept that the CD5^+^ B-1a cell pool is largely, albeit not exclusively, fetal and neonatal derived (28). This conclusion was recently further underscored by the demonstration of a developmental switch between fetal and post-natal development, regulated by the transcription factor Lin28b that significantly affected B-1 cells (29, 30). The studies showed that the expression of Lin28b induces a regulatory network of transcriptional regulators that support the development of B-1a cells. In its absence, B-1a cell populations are greatly reduced, while forced overexpression of Lin28b in adult bone marrow precursors enhances B-1a cell output in adulthood (29, 30). In the latter case, BCR repertoire differences compared with B-1a cells generated from fetal precursors were noted (30), however, suggesting that other signals regulate development and/or selection of these cells.

The lack of sustained *de novo* B-1 cells development beginning from a few weeks after birth was first demonstrated by Lalor et al. (25). It can be exploited experimentally by transferring peritoneal cavity-derived B-1 cells into neonatal mice rendered B cell-deficient by allotype-specific anti-IgM antibody treatment (24, 31). Once recipient mice reach 6 weeks of age, discontinuation of antibody treatment will lead to the reemergence of bone marrow-derived B-2 cell populations, but only few B-1 cells. In that manner, one can generate chimeras in which B-1 cells and their Ig are marked by allotype, or lack or express certain genes only in one of the B cell compartments. Given that B-1 cells are maintained throughout life by self-renewal, i.e., continuous turnover, it will be important to explore the effects of aging on their functionality. Indeed, recent studies suggest alterations to these populations in the aging animals (32). Whether this affects primarily the production of natural IgM, antigen-induced responses of B-1 cells, or both will be an important future target for study.

Thus, the B-1 cell pool of adult mice is likely shaped by distinct waves of B-1 cells that develop from distinct precursors: the first wave of extra-hematopoietic yolk sac B-1 precursors that populate the fetal liver until about E15.5; the second wave of fetal liver precursors that presumably dominates the B-1 cell pool at birth; and the third set in the bone marrow that gives rise to B-1 cells developing during the first few weeks of life (33). All waves are expected to modulate the B-1 cell pool. An unanswered question is to what extent these distinct waves generate B-1 cells of different repertoires, tissue distribution, functionality, and/or lifespan.

### Natural IgM Regulates B Cell Development

Recently, we demonstrated that mice unable to generate secreted (s)IgM contain few B-1 cells in the body cavities, while spleen and bone marrow B-1 cell populations appeared largely unchanged (34). B-2 cell development was also significantly affected. These studies were in apparent contrast to earlier reports that suggested the presence of increased B-1 cell frequencies in the peritoneal cavity of sIgM^−/−^ mice (35, 36), which usually make about 60% of B cells at that site. The discrepancy is explained by our findings that these mice harbor large numbers of anergic, CD5^+^ conventional B cells in both spleen and peritoneal cavity, which due to their expression of CD5 were misidentified as B-1a cells. These anergic CD5^+^ B cells are distinct, however, in that they are CD19^int^, B220^hi^, and CD43^−^, in contrast to the CD19^hi^, B220^lo^, and CD43^+^ B-1a cells (34).

Approximately 1.5–2% of B cells in the spleen of commonly used inbred mouse strains are B-1 cells (37). Interestingly, asplenic Hox11^−/−^ mice were shown previously to have reduced numbers of peritoneal cavity B-1 cells (38) and splenectomy resulted in a reduction of already established peritoneal cavity B-1a cell pools over time (38, 39). Since the spleen is an important tissue source of natural IgM (40, 41) and the Hox11^−/−^ mice showed reductions in serum IgM levels (38), it is unclear whether there is a precursor–offspring relationship between spleen and peritoneal cavity B-1 cells or whether the reductions in sIgM indirectly affected B-1 cell development/expansion.

Our findings, demonstrating alterations in B-2 cell development as early as the bone marrow pre-B cell stage when mice lack secreted IgM, are difficult to reconcile with previous suggestions that the B cell defects seen in the absence of natural IgM are due to lack of apoptotic cell clearance and other “housekeeping functions” attributed to IgM (36, 42). While this could explain the emergence of autoreactive, including anergic, B cells, it is hard to see how this would affect pre-B cell selection. Furthermore, mice that lack the Fc receptor for IgM (FcμR) have about twice the amount of natural serum IgM, yet they develop autoantibodies similar to sIgM^−/−^ mice (43). Based on these and other findings, we suggest that natural IgM regulates B cell development by functions that are independent of its role in the removal of cell debris and autoantigens, but rather by yet to be discovered mechanisms that directly regulate B cell development.

Irrespective of the mechanisms, the findings of a dependency of normal B cell development on natural IgM indicates that the earliest waves of B-1 cells produces the natural IgM that allows normal B-1 and B-2 cell development to commence. The fact that peritoneal cavity B-1 cells, but not splenic B-1 cells, are affected by the lack of IgM further suggests that peritoneal cavity B-1 cells development is dependent on distinct signals and may occur later, after natural IgM production has been initiated in the murine fetus, as IgM, in contrast to IgG, does not effectively cross the placenta (44, 45). Indeed, while small numbers of B-1 cells, including IgM-secreting cells, are detectable before birth in the fetal liver and at birth in the mouse spleen, peritoneal cavity B-1 cells accumulate slowly and not until about 1–2 weeks after birth and the accumulation of B-1 cells in peritoneal and pleural cavity, but not the spleen, are dependent on secretion of the “follicular” homing chemokine CXCL13 (46).

### Repertoire Development of Spleen and Body Cavity B-1 Cells

It is of note that the repertoire of B-1a cells in the peritoneal cavity is distinct from that of the spleen. This is exemplified by measuring frequencies of B-1a cells that bind to liposomes containing phosphotidyl choline (PtC) by FACS, many of which are encoded by IgHV11 (47). While the splenic compartment harbors only about 1–2% of PtC binders among B-1a cells, that frequency is approximately 10% in the peritoneal cavity (48). In mice lacking sIgM, frequencies of PtC binders in the peritoneal cavity and mRNA for IgHV11 are greatly diminished (34), suggesting that B-1a cells with that specificity emerge (or expand) later and then preferentially home to the body cavities.

A recent comprehensive RNA sequencing study on pooled RNA from FACS-purified B-1a and B-2 cells in spleen and peritoneal cavity at various ages of mice further supported previous findings of a B-1 cell B cell receptor (BCR) repertoire that is clearly distinct from that of the B-2 cell pool (49). The B-1 cell repertoire is enriched for self-reactivity, and there is a preponderance of Ig-regions that lack N-region insertions, again supporting the conclusion that B-1 cells are generated before birth, when the enzyme TdT is not yet expressed, and thus does not facilitate inclusions of N-regions during V-D-J recombination. However, both earlier single-cell PCR experiments (50, 51) and the more recent studies (32, 49) demonstrated that the repertoire of B-1a cells is broader, more diverse and not as devoid of N-region insertions as originally anticipated.

The recent B-1a cell repertoire studies support earlier conclusions, which had suggested that the repertoire of the B-1a cell pool is unaffected by foreign antigen exposure. Germ-free mice were shown to have a similar B-1a cell repertoire than that of mice held under SPF housing conditions (49). Similarly, germ-free mice have similar levels of natural IgM and numbers of natural IgM-producing cells in spleen and bone marrow than SPF mice (40). This is not, however, because natural IgM production or the B-1a cell pools are already fully developed at birth. Neonatal mice have very few IgM-secreting cells, few B-1 cells and their serum IgM levels increase dramatically during the first few weeks of life. This was shown already in the 1970s, when early studies demonstrated that antibody-producing cells specific for phosphoryl choline encoded by the T15 idiotype, later found to be expressed nearly exclusively by B-1a cells, did not appear in the spleen and bone marrow until about 1 week after birth and were absent from the fetal liver (52, 53). Frequencies of these antigen-specific cells, while varying in size between individual mice, were present at overall similar frequencies in germ-free and conventionally housed animals, and the pool size was independent of the genotype of the mothers. Similarly, Yang and colleagues noted that the repertoire of B-1a cells in fetal liver and at birth differed from that of B-1a cells in both peritoneal cavity and spleen at weaning and in adulthood (49). Thus, B-1a cells and natural IgM titers undergo dramatic expansions and changes during the first few weeks after birth. While this timing coincides with the first exposure to microbiota and other environmental antigens, the similarities in B-1a cell BCR repertoires between gnotobiotic and SPF-housed mice suggest that this repertoire and the production of natural IgM are regulated in ontogeny, at least in part, by age-specific factors, which could include the emergence of, or exposure to, other cell types, and/or self-antigens.

### Modulating the Pool of Natural IgM-Secreting Cells

In apparent contrast to these findings, early studies demonstrated that B-1 cells can respond to α1–3 dextran (54). More recent studies by Kearney and colleagues have provided evidence that injection of polysaccharides found on the facultative pathogen, *Aspergillus fumigatus*, as well as on house dust mites, permanently altered the natural IgM repertoire when given during a brief window of development just after birth [summarized in Ref. (55)]. While they could not detect significant changes in serum IgM levels, consistent with a body of work demonstrating the inability of newborns to respond to T-independent antigens, their data suggested instead that permanent changes occur affecting the repertoire of polysaccharide-specific B cells. They further demonstrated that this allowed mice to respond to subsequent antigen exposure more robustly with antibody production and a shift away from harmful allergic humoral responses to support immune protective responses (56, 57). These studies are of significance as they further underscore the crucial effects of IgM on the maintenance of tissue and immune homeostasis. They also provide a potential link to and mechanism in support of the “hygiene hypothesis,” which predicts that exposure to pathogens, at an early age, may prime the immune system in such a way that development of allergic reactions are less likely (55).

Apart from the semantic argument of whether IgM antibodies induced in response to foreign antigen exposure/vaccination should still be considered “natural antibodies,” the more important question is why such foreign antigen-induced changes do not seem to affect the BCR repertoire of B-1a cells. Potential explanations are that these antigen-stimulated cells do not arise from the stimulation of B-1 cells, or that following antigen exposure these cells change their phenotype such that they are no longer detectable as part of the “B-1a cell pool.” It will be of interest to study the emerging antigen-specific B cells in greater detail.

## Tissue-Specific Signals as Modulators of B-1 Cell Functions

Adoptive transfer of adult-derived peritoneal cavity B-1 cells into newborn mice seeds all major B-1 cell niches in spleen, body cavities, and bone marrow, such that frequencies of B-1 cells and the antibody-producing cells, and natural serum IgM levels, are at normal levels compared with non-manipulated mice (24, 41, 54). This includes reconstitution of the recently described population of IgM plasma cells in the bone marrow [Ref. (58); Savage et al., under review^1^], demonstrating their B-1 cell origins. This is quite remarkable, given the functional differences of B-1 cells in peritoneal and pleural cavity versus the spleen, as well as their extensive gene expression differences. It is also in apparent contrast to the above data regarding the differential developmental requirements of spleen and body cavity B-1 cells.

It is possible that B-1 cell populations in the body cavities are heterogeneous, containing distinct B-1 cell subsets that will seed the spleen, bone marrow, or body cavities, respectively. Alternatively, B-1 cells are homogenous but have the plasticity to adapt to tissue-specific signals that induce the phenotypic and functional alterations between B-1 cells in these different locations. Overall, scientific evidence to date seems to support the latter. Weiss and colleagues attempted to address this question by adoptively transferring peritoneal cavity B-1 cells and then measuring expression of a handful of genes they had identified as being differentially expressed by spleen and peritoneal cavity B-1 cells (59). Their data showed that the gene expression profile of the donor cells was dependent on their tissue location and not their tissue of origin, thus supporting the idea of tissue-specific signals modulating the gene expression profile of the transferred B-1 cells. However, single-cell transfer or fate-mapping approaches would be necessary to formally rule out that this is due to cell selection rather than tissue-induced changes.

One long known tissue-induced difference between B-1 cells in body cavity and spleen is their differential expression of the β-2 integrin CD11b. While most peritoneal cavity B-1 cells express CD11b, the integrin appears to be lost rapidly after B-1 cell leave the body cavities and enter lymphoid tissues. The small number of CD11b^−^ B-1 cells in the body cavities appears to identify recent arrivals to that site [reviewed in Ref. (4)]. While the tissue-specific signals that induce expression of CD11b on B-1 cells in the body cavities are unknown, we recently demonstrated one of its functions. Specifically, we showed that CD11b was required for effective homing of B-1a cells from body cavities to draining lymph nodes after infection (60). The lack of CD11b did not affect the emigration of B-1a cells from the body cavities. Instead, it was required for the enhanced accumulation of B-1a cells to the lymph nodes. Given that B-1a cells rapidly lose CD11b expression upon entering secondary lymphoid tissues, it appears that the interaction of CD11b with its ligand enhances the entrance of B-1a cells from the blood or lymphatic vessels into the lymph tissue. Thus, CD11b expression by B-1 cells is actively modulated by tissue-specific and/or inflammatory signals.

The abovementioned repertoire differences in BCR expression between B-1 cells in spleen and body cavity may also suggest the presence of tissue-specific factors that drive B-1 cell clonal expansion and/or selection. In the absence of obvious effects of the microbiota on these changes, tissue-restricted expression of autoantigens may account for the distinct repertoires of B-1 cells in different sites. However, this would then also suggest that the B-1 cell compartments of spleen, bone marrow, and body cavity do not usually interchange much. Yet, when we measured the migration of B-1 cells from the body cavity to other tissues, we found that labeled B-1 cells (labeled either with radioisotopes or fluorescent dyes) rapidly disappeared from these sites and then be found in the blood, indicating that there is continuous circulation of body cavity B-1 cells (60).

Another well explored difference between B-1 cells in body cavities and spleen/bone marrow is the fact that spontaneous “natural” IgM production is largely restricted to cells in spleen and bone marrow (41). An earlier study suggested that body cavity macrophages suppress B-1 cell antibody production *via* production of prostaglandin (61). Indeed, isolation of B-1 cells often leads to a larger production of IgM *in vitro*, than culturing cells in the context of the entire cell populations found in the cavities (41). That data fit well with the lack of spontaneous antibody production by B-1 cells in the body cavities and the fact that in response to stimulation, body cavity B-1 cells rapidly migrate to the environments of the spleen and lymph nodes, where they begin to secrete antibodies in response to innate and possibly antigen-specific stimulatory signals (5, 17, 60, 62–65).

Finally, early studies by Kroese and colleagues demonstrated that nearly half of the IgA-secreting plasma cells in the gut are B-1 cell derived (66, 67). Using allotype-chimeric mice similar to studies described by Kroese et al., we found that in the lung of young adult BALB/c mice about one-third of IgA-expressing cells to be B-1 cells (Baumgarth, unpublished)^2^. Based on *in vitro* and *in vivo* studies, it appears that B-1 cells like their B-2 counterparts require TGFβ-signaling for class-switch recombination to IgA, as TGFβ receptor-deficient mice are devoid of all IgA production (68). It is possible that activation of CD11b on peritoneal cavity B-1 cells drives their accumulation in the mesenteric lymph nodes in a manner similar to the activation described by us for B-1 cells from the pleural cavity and their migration to the mediastinal lymph nodes of the lung (60).

While many questions remain, overall there is strong evidence that the tissue environment contributes to the distinct phenotypes and functions of B-1 cells in each tissue compartment.

## B-1 Cell Responsiveness to Self- and Foreign Antigens

### B-1 Cell Responses to Foreign Antigens

The removal of tissue (body cavity)-specific inhibitory signals as a mechanism of regulation for B-1 cell antibody production is an attractive solution to the conundrum of B-1a cell response regulation, a cell that appears unable to respond to anti-IgM induced BCR cross-linking with proliferation or IgM production *in vitro* (69), yet seems to undergo at least limited clonal expansion in response to some antigen-specific signals *in vivo* (see above). However, this unlikely comprises the entire regulatory network controlling B-1 cell activation. Other signals that are known to induce B-1 cell activation include cytokines, specifically IL-5 and IL-10 (70). Lack of IL-5 or its receptor reduces B-1 cell numbers and natural IgM levels in mice (71, 72), including the levels of the classical T15 idiotype-expressing IgM, which binds oxidized low-density lipoproteins and pneumococcal polysaccharides (73). Overexpression of IL-5 *in vivo* (74, 75), exposure of B-1 cells to IL-5 *in vitro*, and injection of IL-5 into the peritoneal cavity *in vivo* induce secretion of IgM following the migration of B-1 cells to the spleen (70).

Consistent with the role of body cavity B-1 cells as surveyors of organ system health and rapid responders to an infection, injection of LPS (76), or bacteria (65) into the peritoneal cavity induces their rapid migration to lymph tissues, where they differentiate to antibody-producing cells. Activation of B-1 cells in the latter case was shown to be dependent on the innate signaling adaptor MyD88 (65) and thus appears mediated by innate rather than antigen-specific B-1 cell stimuli. Similarly, following respiratory tract infection of mice with influenza infection, we noted a rapid drop of B-1a, but not B-1b cells, in the pleural cavity and a concomitant accumulation of these cells in the regional lymph nodes of the respiratory tract (60). The enhanced infection-induced accumulation, but not the migration itself, was dependent on direct signaling *via* the type I IFNR by pleural cavity B-1a cells. Type I IFNR signaling was shown to activate the β-2 integrin CD11b to a high-affinity state, which can bind its ligands and thereby mediate the transfer of B-1 cells across the endothelium (60).

Importantly, in the latter case, the frequency of influenza-binding B-1 cells among those accumulating in the lymph nodes was not significantly different from those in the spleen before influenza infection (17). Also, B-1a cells that accumulated in the lymph nodes did not incorporate significant levels of BrDU, suggesting that these cells responded to influenza infection by relocation and IgM production in an antigen non-specific manner, rather than clonal expansion (17). Yet, following application of non-mitogenic LPS from *Francisella tularensis*, modest expansion of antigen-specific B-1a cells and even formation of memory B cells seemed to occur (5, 6), suggesting that the lack of B-1a cell expansion is not absolute and also depend on the type of antigen.

While responses to both influenza virus infection and non-mitogenic LPS from *F. tularensis* were shown to primarily activate B-1a cells (5), infections with *B. hermsii* (7), *S. pneumonia* (14), and *Salmonella* (10, 11) were shown to stimulate predominantly or exclusively B-1b. In response to infection with *Borrelia burgdorferi*, we found neither B-1a nor B-1b cells to respond in any significant fashion (Hastey et al., in preparation)^3^. What determines this differential responsiveness to antigens and pathogens is unexplored. Possible explanations include the BCR repertoire (50, 51, 77), i.e., differential antigen-specificity among each B cell subset, the site of infection/injection, the quality of the induced innate responses, or other factors. Addressing this question will be important to gain a more complete understanding of the signals that induce B-1 cell responses.

### B-1 Cells and Self-Antigen Recognition

A final consideration for regulation of IgM production by B-1 cells is their responsiveness to self-antigens. Elegant studies by Hayakawa and colleagues demonstrated that the lack of self-antigen expression (in that case the T cell-expressed antigen “Thy-1”) caused a failure to develop self-reactive B-1a cells to that specificity (78), usually a normal component of the B-1a cell repertoire of wild-type mice (79). This data provided the strongest evidence to date that the B-1a cell repertoire is selected for binding to self. Consistent with a positive selection event being necessary for the development of B-1a cells are data indicating that signals that enhance BCR-signaling usually cause increased B-1 cell pools, while mutations reducing BCR-signaling reduce such pools. Examples are deficiencies in the coreceptor CD19 or signaling molecule Btk, causing reduction of B-1a cell numbers, and deficiencies in coinhibitory molecules, such as CD72 increasing these pools [summarized in Ref. (3)]. Thus, positive selection and BCR-signaling appear to be critical elements of B-1a cell development. This is consistent with the resulting B-1 repertoire, which is clearly skewed toward the recognition of self-molecules; particularly those expressed when tissues are altered, stressed, or senescent. Examples are oxidized low-density lipoproteins and antigens expressed by dead and dying cells. The binding of natural IgM to such antigens is thought to fulfill “housekeeping” functions by opsonizing these antigens for removal by phagocytic cells, such as macrophages, while avoiding or actively inhibiting inflammation (80). In certain disease states, however, the generation or accumulation of these antibodies correlates with disease. A prominent example is reperfusion injury, where the binding of natural IgM to tissues stressed or damaged by hypoxia following blood flow disruption is thought to activate the complement cascade and cause tissue injury (81–83).

The recently published comprehensive repertoire analysis conducted on B-1a cells confirmed and expanded previous studies, which together showed differences in the B-1a repertoire based on the age of the host (49). The study by Yang and colleagues reported quite dramatic alterations in the repertoire of spleen and peritoneal cavity B-1a cells, driven by the strong and unequal expansion of certain clones, right after weaning (3 weeks of age) (49). Since alterations in the microbiota were excluded as a potential cause, because similar changes were observed also in gnotobiotic mice, the data might suggest that alterations in, or exposure to certain self-antigens could be causing these repertoire shifts. However, other causes cannot be ruled out, in particular the potential influence of food antigens. The timing of these repertoire changes appears to occur later than the time window identified by Kearney and colleagues by which the B-1 repertoire to certain polysaccharides is amenable to change (55). The fact that B-1 cells have a phenotype similar to activated cells: they express CD43, lack expression of CD23, and have low expression of CD45R (B220) and IgD, could further support the idea that their arrival in the peritoneal cavity and clonal outgrowth is the result of encounter with self-antigens.

### Natural IgM Production and B-1 Cell Repertoires Are Independently Regulated

It is important to distinguish between changes in the repertoire of the overall B-1 cell pool and the extent to which these changes affect the repertoire of the natural IgM-producing cells and thus the serum IgM specificities. Natural IgM-producing cells are distinct from peritoneal cavity B-1 cells and represent only a small subset of B-1 cells in the spleen, currently not distinguishable from non-secreting cells with surface markers. Although a small subset of CD19^+^ CD43^+^ B-1 cells expresses CD138 (84), these are not the major natural IgM producers in the spleen (Savage et al., under review)^1^. Therefore, it should not be assumed that the identified repertoire shifts within the B-1a cell pool necessarily affect the specificity of the secreted natural IgM. It will be important to identify and characterize the B-1 cell-derived natural IgM-producing cells in ontogeny and to compare their repertoire to that of the non-secreting B-1 cells.

The extent to which the secreted antibody repertoire is affected by age and/or antigen exposure is an important question, as this could have implication for immune defense and tissue homeostasis. Indeed, Holodick and colleagues recently provided evidence that IgG-depleted serum from very old mice lost its protective capacity for protection from *S. pneumoniae* infection compared with the same serum preparation from young mice (32). Thus, indicating that both B-1a cell repertoires and secreted IgM repertoires, or their effector functions, can shift with age. What underlies such shifts remains unresolved, given that much of the B-1 cell pool is maintained mainly through self-renewal rather than *de novo* development of B-1 cells, with bone marrow output although not completely abrogated, rather greatly constrained (23). Furthermore, although Patel and Kearney found that immunization during the first few days after birth can alter B-1 cell repertoires, they found no evidence for alterations in the natural IgM serum pool (57), again suggesting that B-1 cell repertoires and natural IgM production are independently regulated and differentially affected by innate and antigen-induced signals.

## Conclusion

The heterogeneity of the B-1 cell precursors, including extra-hematopoietic origins, ontogenetic, and tissue-specific effects, that seem to act on these cells and the high degree of functional plasticity and ability to respond to self- and inflammatory signals generates a diverse set of B-1 cells. Some have effector functions such as IgM secretion or cytokine production. Others, particularly those in the body cavities, appear to be poised to function a natural memory compartment and rapid response system that responds to innate signals with rapid relocation and differentiation.

There are some striking similarities between B-1 cells and macrophages in terms of their developmental origins and effector functions. Not because of a shared ontogeny or phenotype, but rather because of their ontogenic and functional plasticity. Macrophages and B-1 cells both develop in waves from extra-hematopoietic and hematopoietic stem cell precursors (21). They both seed various tissues and differentiate based, at least in part, in response to tissue-specific signals (85). Whether some B-1 cells may also differentiate to become tissue restricted, such as microglia, alveolar macrophages, or other tissue-specific macrophages, remains to be studied. Both cell types also respond rapidly to innate signals with relocation and on-site differentiation. And finally, both cell types fulfill important “housekeeping functions,” in order to maintain tissue homeostasis and to facilitate tissue repair (86). A better understanding of the plasticity and the heterogeneity among B-1 cells will be crucial to better understand the principles underlying their functions and responses.

## Author Contribution

NB conceived and wrote the manuscript.

## Conflict of Interest Statement

The author declares that the research was conducted in the absence of any commercial or financial relationships that could be construed as a potential conflict of interest.

## Footnotes

^1^Savage, H.P., Yenson, V.M., and Baumgarth, N. (2016). Blimp-1 dependent and independent production of natural Ig by B-1 cells and B-1-derived plasma cells. submitted.

^2^Baumgarth, N. (2016). Unpublished observation.

^3^Hastey, C. J., Elsner, R. A., Olsen, K. J., Tunev, S. S., Escobar, E. D., Barthold, S. W., et al. (2016). Borrelia burgdorferi infection induced-IgM controls bacteremia but not bacterial dissemination or tissue burden. in preparation.
